# The association of chronic kidney disease complications by albuminuria and glomerular filtration rate: a cross-sectional analysis 

**DOI:** 10.5414/CN107842

**Published:** 2013-07-27

**Authors:** Gautham Viswanathan, Mark J. Sarnak, Hocine Tighiouart, Paul Muntner, Lesley A. Inker

**Affiliations:** 1 Division of Nephrology, Department of Medicine, Tufts Medical Center, Boston, MA, and; 2 Division of Nephrology, Department of Medicine, University of Alabama at Birmingham, Birmingham, AL, USA

**Keywords:** albuminuria, chronic kidney disease, complications, glomerular filtration rate

## Abstract

Background: Albuminuria is strongly associated with future risk for cardiovascular and kidney outcomes, and has been proposed to be included in the classification of chronic kidney disease (CKD) along with glomerular filtration rate (GFR). Few data are available on whether albuminuria is associated with concurrent complications of CKD. Methods: A cross-sectional analysis of 1,665 participants screened for the Modification of Diet in Renal Disease (MDRD) study was performed to examine the association between albuminuria (determined using urine albumin-creatinine ratio (ACR)) and measured GFR (determined using urinary clearance of iothalamate) with anemia, acidosis, hyperphosphatemia, and hypertension. Results: Mean GFR (± SD) was 39 ml/min/1.73 m^2^ (± 21) and the median (25^ ^– 75^th^ percentile) ACR was 161 (38 – 680) mg/g. In multivariable models adjusted for age, sex, race, kidney disease etiology, and GFR, higher ACR levels were not associated with any complication. For example, comparing ACR > 300 mg/g vs. < 30 mg/g, the prevalence ratio (95% CI) for anemia was 0.98 (0.81 – 1.20), acidosis 1.13 (0.86 – 1.48), hyperphosphatemia 1.69 (0.91 – 3.17), and hypertension 1.04 (0.97 – 1.12). Lower levels of GFR were associated with all complications. For example, GFR levels < 30 ml/min/1.73 m^2^ vs. GFR levels 60 – 89 ml/min/1.73 m^2^ were associated with prevalence ratios (95% CI) of anemia 4.35 (3.18 – 5.96), acidosis 5.31 (3.41 – 8.29), hyperphosphatemia 23.8 (7.71 – 73.6), and hypertension 1.21 (1.10 – 1.32). Conclusions: Albuminuria is not associated with complications after controlling for GFR in patients younger than 70 years of age with non-diabetic CKD and GFR less than 90 ml/min/1.73 m^2^ and thus would not affect clinical action plans for decisions regarding evaluation and treatment of complications in similar populations.

## Introduction 

Chronic kidney disease (CKD) is a major health problem with an increasing incidence and prevalence. Additionally CKD is associated with poor outcomes. The National Kidney Foundation-Kidney Disease Outcomes Quality Initiative (NKF-KDOQI) guidelines for the evaluation, classification, and stratification of risk of CKD defines CKD by glomerular filtration rate (GFR) < 60 ml/min per 1.73 m^2^ or the presence of kidney damage (most commonly by the level of albuminuria) for 3 or more months, and classifies it by the level of GFR [1]. The guidelines include stage-specific clinical action plans to guide clinicians’ evaluation and management of patients with CKD. The staging system has been criticized as it does not provide sufficient information about prognosis, leading to unnecessary investigations, referrals, cost, and patient anxiety [2, 3, 4]. 

Studies have consistently demonstrated that albuminuria is a risk factor for mortality, cardiovascular outcomes, and progression of CKD, independent of GFR [5, 6, 7, 8]. Based on these data, a recent Kidney Disease: Improving Global Outcomes (KDIGO) Controversies Conference recommended revision of the CKD staging system, such that CKD be classified by both level of albuminuria and GFR [9]. Thus far, most studies of albuminuria have focused on its association with future events (e.g., end-stage renal disease, cardiovascular disease, and mortality) [10, 11, 12, 13]. Few data, however, have been published on whether albuminuria is associated with concurrent complications of CKD similar to lower levels of GFR, which is relevant in establishing a clinical action plan and guiding physicians in their decision making and management at a particular patient encounter. We evaluated whether albuminuria is associated with concurrent complications of CKD similar to lower levels of GFR in participants screened for enrollment in the Modification of Diet in Renal Disease (MDRD) Study. We hypothesized that higher levels of albuminuria would be associated with an increased prevalence of hypertension, anemia, hyperphosphatemia, and acidosis, and that these associations would persist despite adjustment for kidney disease etiology and level of GFR. We also evaluated if these associations would be modified by the level of GFR. 

## Subjects and methods 

### Study population 

The MDRD study was a randomized, controlled trial of patients with reduced GFR, predominantly secondary to non-diabetic glomerular disease, tubulo-interstitial disease and polycystic kidney disease [14]. The goal of the study was to evaluate the effects of dietary protein restriction and strict blood pressure control on the progression of kidney disease. Details of the screening and enrollment procedures have been published previously [15, 16, 17]. Briefly, entry criteria for the screening phase included age between 18 and 70 years, serum creatinine of 1.2 – 7.0 mg/dl in women, 1.4 – 7.0 mg/dl in men, or creatinine clearance less than 70 ml/min/1.73 m^2^. Exclusion criteria included pregnancy, Type 1 diabetes, insulin-dependent Type 2 diabetes, glomerulonephritis due to autoimmune diseases such as systemic lupus erythematosus, obstructive uropathy, renal artery stenosis, proteinuria greater than 10 g/d, mean arterial pressure greater than 125 mmHg, and prior kidney transplantation. A total of 2,507 potential participants were screened, and 1,795 met the criteria and were invited for participation into the baseline phase to determine eligibility for the trial. Of the 1,795 participants, 1,665 had data on all the variables of interest for our analysis and are included in the current cross sectional analysis of albuminuria and GFR with concurrent complications of CKD. The investigational review board at Tufts Medical Center, Boston, MA, USA, approved the study. 

### Exposure variables 

**Urine protein**

A 24-hour urine protein excretion was obtained during the MDRD Study screening visit. However, since national and international guidelines, including the National Kidney Disease Education Program (NKDEP), currently recommend use of urine albumin to creatinine ratio (ACR) to detect and evaluate CKD and it is currently the most commonly used measure of urine protein excretion in clinical practice and in research studies, our primary analysis was performed using ACR [18, 19, 20]. The 24-hour urine protein excretion was converted to ACR using the relationship observed in the Irbesartan in Diabetic Nephropathy Trial (IDNT) where ACR = 0.396 × (urine protein)^–0.07^ × 2.45 if female × (urine protein^–0.07^) if female] [21]. We categorized ACR into clinically meaningful categories; < 30 mg/g (normoalbuminuria), 30 – 299 mg/g (microalbuminuria) and > 300 mg/g (macroalbuminuria) [22, 23]. 

**GFR**

GFR was measured using urinary clearance of ^125^I-iothalamate, as has been previously described [15]. Based on the NKF guidelines, GFR was categorized into 3 categories: GFR 60 – 89, 30 – 59 and < 30 ml/min/1.73 m^2^ [1]. Participants with GFR ≥ 90 ml/min/1.73 m^2^ were excluded due to the small number of individuals with this level of GFR. Participants with a GFR of 15 – 29 ml/min/1.73 m^2^ and GFR < 15 ml/min/1.73 m^2^ (CKD Stage 4 and 5, respectively) were combined into one category due to the small number of participants in Stage 5. Since the entry criteria in the screening phase required creatinine clearance of < 70 ml/min/1.73 m^2^, GFR category 60 – 89 essentially consisted of patients with GFR 60 – 70 ml/min/1.73 m^2 ^only. 

**Other variables**

Kidney disease was initially classified into 23 etiologic categories based on history, biopsy, and physician diagnosis but for this analysis it was collapsed into 3 broader categories as previously defined [24]: glomerular disease, polycystic kidney disease (PKD), and others. Diabetes was defined based on review of medical records or patient history [16]. 

### Outcome variables 

The definitions of anemia, acidosis, and hyperphosphatemia were based on accepted cut-offs for patients with CKD [24, 25, 26, 27]. Anemia was defined as hemoglobin less than 12 g/dl for women and less than 13.5 g/dl for men; acidosis as serum bicarbonate level less than 22 mmol/l; and hyperphosphatemia as phosphorus level ≥ 4.5 mg/dl. Study participants were classified as “hypertensive” or “not hypertensive” based on patient history and use of antihypertensive drugs [24]. 

### Statistical analysis 

We summarized clinical characteristics according to ACR categories using proportions for categorical variables and means and standard deviations (SD) for normally distributed continuous variables or medians and the 25^th^ – 75^th^ percentiles for skewed variables. The trends across the ACR categories for the characteristics were tested using Spearman rank correlation for continuous variables and Cochran Armitage trend test for categorical variables. 

We first explored the optimal functional form for ACR using generalized additive models of log ACR for association of each outcome. Since we did not find any non-linear relationships between albuminuria and any of the complications we used the categories of ACR described above in the primary analysis. 

We used log-binomial regression to evaluate the association between ACR category and GFR category, separately, to each of the complications, using ACR < 30 mg/g and GFR 60 – 89 ml/min/1.73 m^2^, as the reference groups. We wanted to express results using prevalence rations as the number of individuals with specific complications was high, and under this circumstance prevalence ratios as obtained by log-binomial regression are preferable. Models were initially unadjusted, but sequential models adjusted for age; age, sex, race, and GFR (or ACR); and age, sex, race, kidney disease etiology, and GFR (or ACR). P-values for linear trend were calculated by modeling category of ACR and GFR as continuous variables in the log-binomial models. We then analyzed the prevalence of the complications across the joint distribution of the ACR categories and GFR categories. We tested the interaction of ACR and GFR categories for modification of the effect of ACR on the complications by level of GFR. 

In sensitivity analyses, we repeated the analyses using proteinuria in place of ACR, categorizing proteinuria by < 200 mg/24 h, 200 – 999 mg/24 h and ≥ 1,000 mg/24 h [1, 28]. We also modeled ACR and GFR as continuous variables. Since ACR was not distributed normally, we used log transformed ACR. 

Statistical analyses were performed using SAS (version 9.2). A two-tailed p-value of < 0.05 was considered statistically significant. 

## Results 

The baseline characteristics of the study population according to the ACR categories are listed in Table 1. The mean ± SD age of the cohort was 51 ± 13 years, 60% of patients were male, 80% were white, and 6% had diabetes. Also, 32% of patients had glomerular disease, 22% had polycystic kidney disease, and 46% had tubulointerstitial, hypertension or other causes of kidney disease. The mean GFR (± SD) was 38 ± 19 ml/min/1.73 m^2^, median (25^th^ – 75^th^ percentiles) for ACR and proteinuria were 161 mg/g (38 – 680) and 320 mg/24 h (60 – 1,400), respectively. Patients with higher ACR were younger, less likely to be white and male and had lower GFR (p-value < 0.001 for all). A higher proportion of glomerular disease was noted in the higher ACR category. 

The prevalence of anemia, acidosis, hyperphosphatemia, and hypertension was 43%, 31%, 16%, and 81%, respectively. There was a graded increase in the prevalence of anemia, acidosis, hyperphosphatemia and hypertension with increasing levels of albuminuria (Table 2). After age adjustment, there was a significant association between higher ACR levels with higher prevalence ratios for each complication (p-trend < 0.001 for each complication) (Table 3). After adjustment for age, sex, race, GFR, and kidney disease etiology, higher ACR levels were not associated with any complications. 

The prevalence of anemia, acidosis, hyperphosphatemia, and hypertension increased with decreasing GFR (p-trend < 0.001 for each complication) (Table 4). In age-adjusted models and multivariable-adjusted models, lower GFR levels were significantly associated with higher prevalence ratios for anemia, acidosis, hyperphosphatemia, and hypertension (p-trend < 0.001 for each complication) (Table 5). 

**Figure 1.**
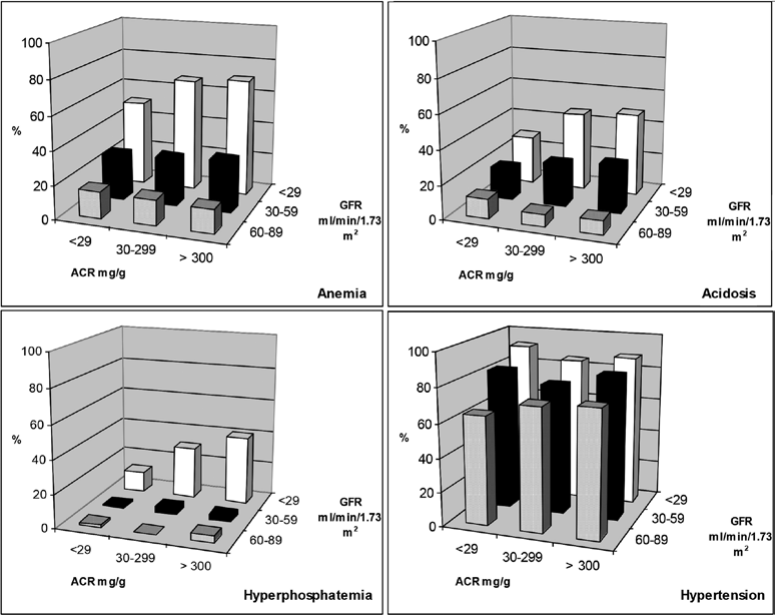
Prevalence of CKD complications stratified by level of albumin-creatinine ratio (ACR) and glomerular filtration rate (GFR). 3-D columns showing the prevalence of CKD complications stratified by ACR and GFR. The different shades represent different GFR categories; Grey, black, and white represents GFR 60 – 89, 30 – 59, and < 29 ml/min/1.73 m^2^, respectively. ACR = albumin-creatinine ratio; GFR = glomerular filtration rate.

Figure 1 and Supplemental Table 1 show the prevalence of CKD complications stratified by the joint distribution of ACR categories and GFR categories. There was a strong association between GFR and each complication within each ACR category. In contrast, the associations between ACR and complications were not consistently present within GFR strata. In patients with GFR < 30 ml/min/1.73 m^2^, but not 30 – 59 or 60 – 89 ml/min/1.73 m^2^, increasing levels of albuminuria were associated with a higher prevalence of anemia, hyperphosphatemia, and acidosis. However, the interaction between ACR categories and GFR categories was not significant (p-value > 0.2) for each outcome. 

In sensitivity analysis, the results using proteinuria were similar to those using ACR (Supplemental Table 2). For example, in age-adjusted analyses, higher levels of proteinuria was associated with anemia, acidosis, hyperphosphatemia, and hypertension (each p-value < 0.001), while in models adjusted for age, sex, race, GFR, and disease etiology, proteinuria greater than 1,000 mg/24 h was associated with a higher prevalence ratio (95% CI) of hyperphosphatemia 1.34 (1.00 – 1.79) but not with the other complications. The results were similar to the primary analysis when repeated using albuminuria and GFR as continuous variables (data not shown). 

## Discussion 

In this study of participants with predominantly non-diabetic CKD, after adjustment for age, race, sex, GFR, and etiology of kidney disease, there was no association between ACR and concurrent complications of CKD, and ACR did not appear to modify the effect of GFR on complications. In contrast, consistent with previous reports, we observed strong associations between lower levels of GFR and each complication studied [1, 29]. These results have implications in the management of CKD and in the consideration of clinical action plans associated with combining both albuminuria and GFR in CKD classification. 

Many studies have demonstrated that albuminuria is an independent predictor of mortality, cardiovascular outcomes, and progression of CKD [5, 7, 30, 31]. For example, in a long term follow-up of the NHANES III participants, the presence of albuminuria > 300 mg/g was more strongly associated with mortality than lower levels of eGFR [6]. Similarly, in a meta-analysis of 21 cohorts with 105,872 participants, estimated GFR ≥ 60 ml/min/1.73 m^2^ and ACR ≥ 10 mg/g were independently associated with all-cause and cardiovascular mortality risk in the general population [32]. In a general population study in Italy, albuminuria was associated with hypertension, high serum uric acid levels, and cardiovascular disease, but not hypokalemia, hyperphosphatemia, or hypocalcemia suggesting that albuminuria might be associated with certain complications, but not all [33]. The key hypothesized mechanism for these associations are that albuminuria is associated with inflammation and vascular endothelial injury, which in turn are associated with adverse outcomes of CKD, including cardiovascular disease and death [5, 34, 35]. As such, albuminuria is of critical value in determining long-term prognosis and has, therefore, been proposed as an additional marker to GFR in CKD classification. 

There are several possible mechanisms by which albuminuria could be also associated with CKD complications. First albuminuria has been associated with systemic inflammation, which could contribute to the development of anemia and hypertension [36, 37]. Positive associations have been present in populations where albuminuria is due to generalized endothelial disease and inflammation [36, 37]. The lack of positive associations in our study may have been due to different mechanisms of albuminuria. In the MDRD study population the magnitude of albuminuria is larger and likely reflects the underlying glomerular injury rather than generalized endothelial dysfunction. In our similar analyses of the NHANES population, there was a significant, although mild to moderate, association between increasing ACR levels and anemia, acidosis, hypoalbuminemia, hyperparathyroidism, and hypertension. The significant associations in that population vs. the MDRD study population described here may be due to the fact that albuminuria was secondary to generalized endothelial dysfunction rather than specific kidney injury. Second, in patients with large amounts of albuminuria, there is likely to be increased glomerular loss of erythropoietin and hormone binding protein such as transferrin contributing to the development of anemia [38]; as well as resulting proximal tubular damage contributing to the development of acidosis [39]. Consistent with this hypothesis, most of the previously reported associations between albuminuria and CKD complications have been noted in patients with nephrotic syndrome where the urinary protein excretion is greater than 3,000 mg/24 hours [38]. As such, the lack of significant findings in our study may be due to the moderate levels of proteinuria in the MDRD study population. Few people with urinary protein excretion above 3 g/day (n = 190) were included in the MDRD Study. It is possible that associations between albuminuria and the complications we studied may be more apparent in populations with more severe levels of albuminuria. While hypertension was analyzed as a complication of CKD, hypertension in and of itself may cause albuminuria through hemodynamic mechanisms. Thus, the absence of an association was unexpected and may have been due to the cross sectional nature of the study. Additionally, the high prevalence of hypertension and anti-hypertensive medication use might have limited our ability to detect the association. The findings in our study have significant implications when considering the issues surrounding the current CKD classification and proposals to include albuminuria along with GFR during classification of CKD severity as well as in the management of CKD complications at a given clinical encounter. These results suggest that while albuminuria will provide information about the long-term risk for adverse outcomes, including development of kidney failure and mortality, it will not provide additional benefit in the evaluation and management of concurrent complications. Thus, when health care professionals encounter patients with CKD who have both a decreased GFR and elevated albuminuria they would have to assess the risks associated with GFR and albuminuria separately. For example, they could use the information on albuminuria for prognostic outcomes and initiate appropriate treatments for reduction in albuminuria such as treatment with renin angiotensin system blockers and target blood pressure levels less than 130/80 mmHg for slowing the rate of progression of non-diabetic kidney disease [40]. Conversely, clinicians could use the level of GFR to guide screening and management of concurrent CKD complications, adjustment of drug dosing, stratifying patients at risk for acute kidney injury when using contrast agents and planning for renal replacement therapy [1, 41, 42]. 

The strengths of this study include the analysis of a large number of individuals from a well-established cohort with moderate-to-severe CKD, availability of measured GFR, detailed ascertainment of confounding variables including CKD etiology, and use of clinically relevant ACR cut-offs that allows for comparability with future studies from other cohorts and thus allow for generalizability of study results to the current era. The study population is restricted mostly to patients with progressive CKD not due to diabetes and therefore is a unique and homogenous population. The cross sectional study design is appropriate as the question being asked refers to point of care in the management of patients with CKD. 

There are several limitations to our study. First, ACR was not measured directly but rather derived from 24-hour urine protein excretion using a relationship observed between ACR and 24-hour protein excretion in the IDNT study, a study of diabetic nephropathy, where albumin is the predominant urinary protein. The MDRD Study, consisted of non-diabetic kidney disease and the total urine protein in this population likely contains in addition to albumin, non-albumin proteins such as β-2 microglobulin, retinol binding protein, and other low-molecular weight proteins [43]. Thus, the relationship observed in IDNT might not be accurate. However, analyses using proteinuria demonstrated similar results to ACR. Second, blood pressure measurement itself was not used as a criterion for defining hypertension since baseline blood pressure levels were recorded with patients taking their usual antihypertensive medication. Third, we were unable to analyze the association between albuminuria and other CKD complications such as secondary hyperparathyroidism, malnutrition and inflammation as these parameters were not measured in the MDRD study. Fourth, possibly interactions between albuminuria and GFR were not significant due to small sample sizes in some of the subgroups. Finally, the results are generalizable only to patients younger than 70 years of age with non-diabetic CKD and GFR less than 90 ml/min/1.73 m^2^. A significant proportion of patients have CKD based on proteinuria alone. The results will need to be reproduced in other populations such as diabetic kidney disease and other kidney diseases not represented in the MDRD study. 

In conclusion, ACR does not provide any additional information about CKD complications beyond what is already known with GFR. These findings should be taken into account when considering the inclusion of albuminuria level along with GFR in the development of clinical action plans associated with the proposed new classification of CKD. GFR and albuminuria represent different aspects of kidney disease and management decisions should be based on their individual association with complications and ability to predict adverse outcomes and should be assessed separately for their clinical utility. 

## Acknowledgment 

Research Support: This research is supported by a National Kidney Foundation/Amgen Research Fellowship grant to Dr. Viswanathan. Dr. Inker is supported by a grant K23-DK081017. Dr. Sarnak is supported by K24DK078204. 

## Conflict of interest

None declared. 

**Table 1.** Characteristics of MDRD participants, overall, and by albumin-to-creatinine ratio (ACR) categories.

Characteristics	Overall	ACR mg/g			
		< 30	30 – 299	> 300	p-trend
Total number of patients, n (%)	1,665	307 (18.4)	696 (41.8)	662 (39.8)	
Age (y)	51 (13)	56 (11)	50 (12)	49 (13)	< 0.001
Men (%)	60	84.0	46.8	64.2	0.0011
White (%)	80	86.6	82.8	74.8	< 0.001
Body mass index (kg/m^2^)	27 (5)	28 (4)	27 (5)	28 (5)	0.22
Diabetes (%)	6	3	4	9	< 0.001
Coronary artery disease n (%)	148 (8)	33 (10.7)	53 (7.6)	62 (9.4)	0.23
Serum albumin (g/dl)	4.0 (0.4)	4.2 (0.3)	4.1 (0.3)	3.8 (0.4)	< 0.001
Serum creatinine (mg/dl)	2.3 (1.2)	1.8 (0.7)	2.2 (1.0)	2.8 (1.3)	< 0.001
Potassium (mEq/l)	4.3 (0.6)	4.2 (0.5)	4.3 (0.6)	4.4 (0.6)	< 0.001
GFR (ml/min/1.73 m^2^)	38 (19)	48 (17)	39 (19)	31 (17)	< 0.001
GFR 60 – 89 n (%)	223 (13.4)	69 (22.5)	104 (14.9)	50 (7.6)	< 0.001
GFR 30 – 59 n (%)	769 (46.2)	189 (61.6)	343 (49.3)	237 (35.8)	< 0.001
GFR < 29 n (%)	673 (40.4)	49 (16)	249 (35.8)	375 (56.7)	< 0.001
Urine total protein mg/d^a^	320 (60 – 1,400)	40 (30 – 50)	140 (80 – 310)	1,890 (1,060 – 3,280)	< 0.001
Urine ACR mg/g^a^	161 (38 – 680)	16 (12 – 23)	72 (45 – 157)	869 (529 – 1,565)	< 0.001
Kidney disease etiology, n (%)					
PKD	368 (22.1)	75 (24.4)	239 (34.3)	54 (8.2)	< 0.001
GN (GN, hereditary and DM)	539 (32.4)	26 (8.5)	150 (21.6)	363 (54.8)	< 0.001
Others	758 (45.5)	206 (67.1)	307 (44.1)	245 (37.0)	< 0.001
Hemoglobin (g/dl)	13.1 (1.9)	14 (1.6)	13 (1.8)	12.8 (2.16)	< 0.001
Bicarbonate (mEq/l)	23.3 (3.8)	24.4 (3.4)	23.4 (3.8)	22.5 (3.9)	< 0.001
Phosphorus (mg/dl)	3.8 (0.8)	3.4 (0.6)	3.8 (0.7)	4.0 (0.9)	< 0.001
Systolic blood pressure (mmHg)	133 (18)	130 (18)	130 (18)	138 (19)	< 0.001
Diastolic blood pressure (mmHg)	81 (11)	80 (10)	81 (11)	84 (11)	< 0.001

Means and SD unless mentioned, ^a^Median (25 – 75^th^ percentile). n = number; GFR = glomerular filtration rate; ACR = albumin creatinine ratio; PKD = polycystic kidney disease; GN = glomerulonephritis; DM = diabetes.

**Table 2.** Prevalence of complications across albumin-to-creatinine ratio (ACR) categories.

	Overall	ACR mg/g			p-trend
		< 29	30 – 299	> 300	
Total number of patients, n (%)	1,665 (100.0)	307 (18.4)	696 (41.8)	662 (39.8)	
Anemia (%)	42.8	28.7	40.5	51.8	< 0.001
Acidosis (%)	31.1	18.6	29.7	38.2	< 0.001
Hyperphosphatemia (%)	18.0	4.2	15.4	27.2	< 0.001
Hypertension (%)	81.7	79.8	78.7	85.8	0.0045

n = number; ACR = urine albumin-to-creatinine ratio.

**Table 3.** Adjusted prevalence ratios of CKD complications associated with level of albuminuria.

Complication	Model	ACR, mg/g			p-trend
		< 29 (n = 307)	30 – 299 (n = 696)	> 300 (n = 662)	
Anemia	Age	1 (ref)	**1.42 (1.16 – 1.73)**	**1.81 (1.49 – 2.20)**	**< 0.001**
	MV	1 (ref)	1.07 (0.88 – 1.29)	1.01 (0.84 – 1.23)	0.76
	MV2	1 (ref)	1.04 (0.86 – 1.26)	0.98 (0.81 – 1.20)	0.99
Acidosis	Age	1 (ref)	**1.55 (1.19 – 2.02)**	**1.97 (1.52 – 2.55)**	**< 0.001**
	MV	1 (ref)	1.12 (0.86 – 1.46)	1.14 (0.88 – 1.48)	0.46
	MV2	1 (ref)	1.13 (0.86 – 1.47)	1.13 (0.86 – 1.48)	0.55
Hyperphosphatemia	Age	1 (ref)	**3.50 (1.99 – 6.13)**	**6.12 (3.53 – 10.61)**	**< 0.0001**
	MV	1 (ref)	1.33 (0.78 – 2.28)	1.51 (0.89 – 2.56)	0.04
	MV2	1 (ref)	1.30 (0.76 – 2.23)	1.48 (0.87 – 2.53)	0.07
Hypertension	Age	1 (ref)	1.02 (0.95 – 1.09)	**1.12 (1.05 – 1.19)**	**< 0.001**
	MV	1 (ref)	0.99 (0.93 – 1.06)	1.03 (0.97 – 1.10)	0.18
	MV2	1 (ref)	0.98 (0.92 – 1.05)	1.04 (0.97 – 1.12)	0.16

Numbers in table are prevalence ratios (95% confidence interval); ACR = urine albumin to creatinine ratio; MV = multivariable adjusted for age, sex, and GFR continuous; MV2 = multivariable adjusted for age, sex, race, diagnostic categories, and GFR continuous. Numbers in **bold** represent statistically significant values.

**Table 4.** Prevalence of CKD complications across glomerular filtration rate categories.

	Overall	GFR ml/min/1.73 m^2^			p-trend
		60 – 89	30 – 59	< 30	
Total number of patients, n (%)	1,665 (100.0)	223 (13.4)	769 (46.2)	673 (40.4)	
Anemia (%)	42.8	15.3	29	67.8	< 0.001
Acidosis (%)	31.1	8.52	24.2	46.4	< 0.001
Hyperphosphatemia (%)	15.6	2.2	3.8	39.5	< 0.001
Hypertension (%)	81.7	69.9	80.2	87.4	< 0.001

n = number; GFR = glomerular filtration rate.

**Table 5.** Adjusted prevalence ratios for CKD complications by level of glomerular filtration rate.

Complication	Model	GFR ml/min/1.73 m^2^			p-trend
		60 – 89 (n = 223)	30 – 59 (n = 769)	< 30 (n = 673)	
Anemia	Age	1 (ref)	**1.92 (1.38 – 2.67)**	**4.47 (3.27 – 6.12)**	< 0.001
	MV	1 (ref)	**1.90 (1.37 – 2.64)**	**4.37 (3.19 – 5.98)**	< 0.001
	MV2	1 (ref)	**1.91 (1.37 – 2.65)**	**4.35 (3.18 – 5.96)**	< 0.001
Acidosis	Age	1 (ref)	**2.92 (1.86 – 4.59)**	**5.54 (3.57 – 8.61)**	< 0.001
	MV	1 (ref)	**2.88 (1.83 – 4.53)**	**5.32 (3.41 – 8.29)**	< 0.001
	MV2	1 (ref)	**2.88 (1.84 – 4.53)**	**5.31 (3.41 – 8.29)**	< 0.001
Hyperphosphatemia	Age	1 (ref)	2.22 (0.67 – 7.31)	**26.64 (8.65 – 82.10)**	< 0.001
	MV	1 (ref)	2.14 (0.65 – 7.08)	**23.88 (7.73 – 73.83)**	< 0.001
	MV2	1 (ref)	2.15 (0.65 – 7.10)	**23.82 (7.71 – 73.60)**	< 0.001
Hypertension	Age	1 (ref)	**1.13 (1.03 – 1.24)**	**1.24 (1.13 – 1.35)**	< 0.001
	MV	1 (ref)	**1.12 (1.02 – 1.23)**	**1.21 (1.11 – 1.33)**	< 0.001
	MV2	1 (ref)	**1.12 (1.02 – 1.23)**	**1.21 (1.10 – 1.32)**	< 0.001

Numbers in table are prevalence ratios (95% confidence interval) GFR = glomerular filtration rate; MV = multivariable adjusted for age, sex, race, and albumin-to-creatinine ratio as a continuous variable; MV2 = multivariable adjusted for age, sex, race, diagnostic categories, and albumin-to-creatinine ratio as a continuous variable. Numbers in **bold** represent statistically significant values.

**Supplemental Table 1.** Prevalence of CKD complications (%) stratified by the joint distribution of ACR categories and GFR categories.

	GFR ml/min/1.73 m^2^	ACR, mg/g		
		< 29 (n = 307)	30 – 299 (n = 696)	> 300 (n = 662)
No. (%) of patients	60 – 89 (n = 223)	69 (4.1)	104 (6.3)	50 (3)
	30 – 59 (n = 769)	189 (11.4)	343 (20.6)	237 (14.2)
	< 29 (n = 673)	49 (2.9)	249 (15)	375 (22.5)
Complication				
Anemia (%)	60 – 89 (n = 223)	15.9	15.4	14.0
	30 – 59 (n = 769)	27.5	28.6	30.8
	< 29 (n = 673)	51.0	67.5	70.1
Acidosis (%)	60 – 89 (n = 223)	11.6	6.7	8.0
	30 – 59 (n = 769)	18.5	24.8	27.9
	< 29 (n = 673)	28.6	46.2	48.8
Hyperphosphatemia (%)	60 – 89 (n = 223)	1.5	0.0	4.0
	30 – 59 (n = 769)	1.1	3.8	2.9
	< 29 (n = 673)	12.2	30.9	40.5
Hypertension (%)	60 – 89 (n = 223)	63.8	72.1	74.0
	30 – 59 (n = 769)	82.5	76.4	83.9
	< 29 (n = 673)	91.8	84.7	88.5

ACR = urine albumin to creatinine ratio.

**Supplemental Table 2.** Age and multivariable adjusted prevalence ratios of CKD complications associated with level of proteinuria.

Complication	Model	Proteinuria mg/24-hour			p-trend
		< 199 (n = 734)	200 – 999 (n = 409)	1,000 (n = 522)	
Anemia	Age	1 (ref)	1.35 (1.16 – 1.57)	1.66 (1.46 – 1.90)	< 0.0001
	MV	1 (ref)	0.93 (0.82 – 1.07)	0.98 (0.86 – 1.11)	0.76
	MV2	1 (ref)	0.93 (0.81 – 1.07)	0.97 (0.85 – 1.12)	0.74
Acidosis	Age	1 (ref)	1.37 (1.14 – 1.66)	1.64 (1.38 – 1.94)	< 0.0001
	MV	1 (ref)	1.03 (0.85 – 1.23)	1.07 (0.89 – 1.27)	0.47
	MV2	1 (ref)	1.02 (0.85 – 1.23)	1.05 (0.87 – 1.28)	0.61
Hyperphosphatemia	Age	1 (ref)	2.49 (1.78 – 3.49)	3.60 (2.65 – 4.88)	< 0.0001
	MV	1 (ref)	1.19 (0.89 – 1.59)	1.34 (1.02 – 1.75)	0.028
	MV2	1 (ref)	1.20 (0.90 – 1.60)	1.34 (1.00 – 1.79)	0.046
Hypertension	Age	1 (ref)	1.04 (0.98 – 1.11)	1.10 (1.05 – 1.16)	< 0.0001
	MV	1 (ref)	0.98 (0.92 – 1.04)	1.01 (0.96 – 1.06)	0.64
	MV2	1 (ref)	0.99 (0.93 – 1.05)	1.02 (0.96 – 1.09)	0.48

MV = adjusted for age, sex, race, and GFR continuous; MV2 = adjusted for age, sex, race, diagnostic categories, and GFR continuous.
